# A review of the Anomaloninae (Hymenoptera, Ichneumonidae, Anomaloninae) from the Ukrainian Carpathians

**DOI:** 10.3897/BDJ.3.e6890

**Published:** 2015-12-21

**Authors:** Anna Nuzhna, Oleksandr Varga

**Affiliations:** ‡ Schmalhausen Institute of Zoology, NAS of Ukraine, Kiyv, Ukraine

**Keywords:** Ichneumonidae, Anomaloninae, Ukrainian Carpathians

## Abstract

**Background:**

The Ukrainian Anomaloninae fauna is relatively poorly known. The presence of large under-collected areas, such as the Carpathians, makes taxonomic and faunistic studies concerning these parasitoids from Ukraine urgently relevant.

**New information:**

Based on our ongoing surveys on the Anomaloninae of the Ukrainian Carpathians, we report here the new distribution records for some species. In total 24 Anomaloninae species, belonging to 8 genera (*Anomalon* Panzer, 1804, *Agrypon* Förster, 1868, *Aphanistes* Förster, 1868, *Barylypa* Förster, 1868, *Heteropelma* Wesmael, 1849, *Perisphincter* Townes, 1961, *Therion* Curtis, 1829, and *Trichomma* Wesmael, 1849), were recorded from the studied region for the first time. Nine species, *Agrypon batis* Ratzeburg, 1955, *A. scutellatum* Hellén, 1926, *Aphanistes gliscens* Hartig, 1838, *A. klugii* Hartig, 1838, *Heteropelma amictum* Fabricius, 1775, *Perisphincter gracilicornis* Schnee, 1978, *Therion giganteum* Gravenhorst, 1829, *Trichomma fulvidens* Wesmael, 1849 and *T. occisor* Habermehl, 1909 were recorded for the first time in Ukraine. Seasonal dynamics and high-altitude zone of Anomaloninae species' distribution are discussed.

## Introduction

Anomaloninae Viereck,1918 (Hymenoptera, Ichneumonidae) is a medium-sized cosmopolitan subfamily represented worldwide (Yu et al. 2012​).

The *s* ubfamily Anomaloninae is divided into two tribes: Anomalonini with one genus *Anomalon* Panzer (parasitoids of Coleoptera larvae (Tenebrionidae and Elateridae) and some Lepidoptera larvae (Noctuidae and Tortricidae) and Gravenhorstiini containing the remaining genera (parasitoids of Lepidoptera larvae) (Schnee, 2014). The main morphological features of the subfamily Anomaloninae are: metasoma slender; propodeum usually coarsely reticulate; fore wing with vein 3*rs-m* absent; hind tarsi of male often swollen.

Unlike many other ichneumonids occuring in wet areas, some Anomaloninae species prefer dry places, such as steppes and forest-steppe regions. The Ukrainian fauna of the subfamily Anomaloninae is still poorly studied and represented just by 30 recorded species (Atanasov 1981, Nuzhna 2013). This paper provides the first list of Anomaloninae species recorded from the Ukrainian Carpathians, which includes some interesting records of quite rare species and new records for the Ukrainian fauna.

## Materials and methods

This study is mainly based on specimens collected by sweep netting and Malaise traps. Sampling was conducted by the second author in various locations of the Ukrainian Carpathians and adjacent territories in 2009-2013. Specimens deposited in the collections of the Schmalhausen Institute of Zoology (Kiyv) and the Vasyl Stefanyk Precarpathian National University (Ivano-Frankivsk) were also studied. Morphological terminology used in the study follows that of Gauld (1991). The photos of the specimens were made using the camera Canon Power Shot A 4050 IS attached to light microscope Olympus SZX10. Some species were examined using Scanning Electron Microscopy (SEM: JEOL JSM-6480LV with a low vacuum option) in Royal Museum for Central Africa (Tervuren, Belgium). Specimens were identified using the keys compiled by Atanasov (1981), Gauld (1976), Gauld and Mitchell (1977), Nuzhna (2013), and Schnee (1978).

## Taxon treatments

### Anomalon cruentatum

Geoffroy, 1785

#### Materials

- **Type status:** Other material. **Occurrence:** recordedBy: O. Varga; individualCount: 1; sex: female; lifeStage: adult; **Location:** country: Ukraine; stateProvince: Transcarpathian region; county: Tyachiv district; locality: Solotvyno; verbatimElevation: 280 m; verbatimCoordinates: 47°57'15.82"N, 23°52'13.95"E; **Identification:** identifiedBy: A. Nuzhna; dateIdentified: 2013; **Event:** samplingProtocol: sweeping; eventDate: 06/23/2013

#### Distribution

Palaearctic and Oriental regions (Yu et al. 2012). Ukraine: all regions (Nuzhna 2010) (Fig. 1).

### Agrypon anomelas

(Gravenhorst, 1829)

#### Materials

- **Type status:** Other material. **Occurrence:** recordedBy: O. Varga; individualCount: 1; sex: male; lifeStage: adult; **Location:** country: Ukraine; stateProvince: Transcarpathian region; county: Rakhiv district; locality: Marmarosy, 12 km SE of Dilove; verbatimElevation: 1400-1500 m; verbatimCoordinates: 47°54'56.55"N, 24°17'43.41"E; **Identification:** identifiedBy: A. Nuzhna; dateIdentified: 2013; **Event:** samplingProtocol: sweeping; eventDate: 6-9.08.2011

#### Distribution

Palaearctic and Oriental regions (Yu et al. 2012). Ukraine: Chernihiv, Kyiv, Lugansk, Mykolaiv, Odesa, Zaporizhzhya regions and Crimea (Nuzhna 2013​)​, Transcarpathian region.

### Agrypon anxium

(Wesmael, 1849)

#### Materials

- **Type status:** Other material. **Occurrence:** recordedBy: O. Varga; individualCount: 1; sex: male; lifeStage: adult; **Location:** country: Ukraine; stateProvince: Ivano-Frankivsk region; county: Bogorodchany district; locality: Mochary, mixed forest, 5 km NE of Bogorodchany; verbatimElevation: 300-350 m; verbatimCoordinates: 48°50'51.17"N, 24°35'26.91"E; **Identification:** identifiedBy: A. Nuzhna; dateIdentified: 2013; **Event:** samplingProtocol: sweeping; eventDate: 09/10/2010
- **Type status:** Other material. **Occurrence:** recordedBy: O. Varga; individualCount: 2; sex: females; lifeStage: adult; **Location:** country: Ukraine; stateProvince: Ivano-Frankivsk region; county: Bogorodchany district; locality: Gorgany, coniferous forest, 10-12 km SW of Stara Guta; verbatimElevation: 1200-1300 m; verbatimCoordinates: 48°33'7.45"N, 24°11'55.33"E; **Identification:** identifiedBy: A. Nuzhna; dateIdentified: 2013; **Event:** samplingProtocol: sweeping; eventDate: 17-19.08.2011
- **Type status:** Other material. **Occurrence:** recordedBy: O. Varga; individualCount: 1; sex: female; lifeStage: adult; **Location:** country: Ukraine; stateProvince: Transcarpathian region; county: Rakhiv district; locality: Sheshul, 6-7 km NE of Kvasy; verbatimElevation: 1400-1500 m; verbatimCoordinates: 48°09'25.05"N, 24°20' 59.53"E; **Identification:** identifiedBy: A. Nuzhna; dateIdentified: 2013; **Event:** samplingProtocol: sweeping; eventDate: 07/09/2011
- **Type status:** Other material. **Occurrence:** recordedBy: O. Varga; individualCount: 2; sex: males; lifeStage: adult; **Location:** country: Ukraine; stateProvince: Transcarpathian region; county: Rakhiv district; locality: Marmarosy, ​12 km SE of Dilove; verbatimElevation: 1400-1500 m; verbatimCoordinates: 47°54'56.55"N, 24°17'43.41"E; **Identification:** identifiedBy: A. Nuzhna; dateIdentified: 2013; **Event:** samplingProtocol: sweeping; eventDate: 6-9.08.2011
- **Type status:** Other material. **Occurrence:** recordedBy: O. Varga; individualCount: 6; sex: females; lifeStage: adult; **Location:** country: Ukraine; stateProvince: Transcarpathian region; county: Rakhiv district; locality: Marmarosy, ​12 km SE of Dilove; verbatimElevation: 1400-1500 m; verbatimCoordinates: 47°54'56.55"N, 24°17'43.41"E; **Identification:** identifiedBy: A. Nuzhna; dateIdentified: 2013; **Event:** samplingProtocol: sweeping; eventDate: 6-9.08.2011

#### Distribution

Palaearctic region (Yu et al. 2012). Ukraine: Chernihiv, Kharkiv, Kyiv, Rivne regions and Crimea (Nuzhna 2013), Ivano-Frankivsk and Transcarpathian regions.

### Agrypon batis

(Ratzeburg, 1855)

#### Materials

- **Type status:** Other material. **Occurrence:** recordedBy: V. Tolkanitz; individualCount: 1; sex: female; lifeStage: adult; **Location:** country: Ukraine; stateProvince: Ivano-Frankivsk region; county: Nadvirna district; locality: Chornogora, pol. Pozhezhevska; verbatimElevation: 1400-1500 m; **Identification:** identifiedBy: A. Nuzhna; dateIdentified: 2013; **Event:** samplingProtocol: sweeping; eventDate: 06/26/1975
- **Type status:** Other material. **Occurrence:** recordedBy: O. Varga; individualCount: 1; sex: female; lifeStage: adult; **Location:** country: Ukraine; stateProvince: Ivano-Frankivsk region; county: Bogorodchany district; locality: Pidhirya; verbatimElevation: 300-350 m; verbatimCoordinates: 48°47'27.47"N, 24°30'44.41"E; **Identification:** identifiedBy: A. Nuzhna; dateIdentified: 2013; **Event:** samplingProtocol: sweeping; eventDate: 05/29/2012

#### Distribution

**Figure 2.**
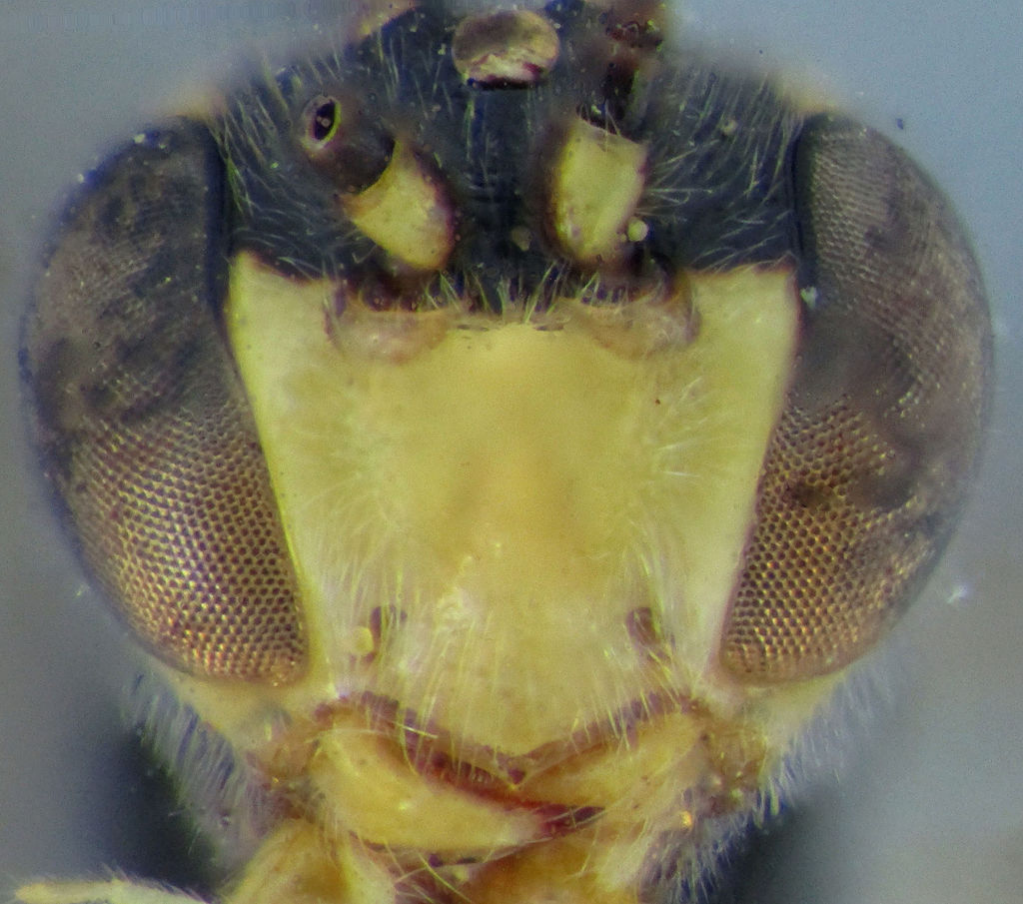
*Agrypon batis* (Ratzeburg, 1855), female, dorsal view of face.

Western Palaearctic region (Yu et al. 2012). Ukraine: Ivano-Frankivsk region. New record for Ukraine (Fig. 2).

### Agrypon clandestinum

(Gravenhorst, 1829)

#### Materials

- **Type status:** Other material. **Occurrence:** recordedBy: O. Varga; individualCount: 1; sex: female; lifeStage: adult; **Location:** country: Ukraine; stateProvince: Ivano-Frankivsk region; county: Nadvirna district; locality: Gorgany, Elmy, coniferous forest, 15 km SW of Yaremche; verbatimElevation: 800-900 m; verbatimCoordinates: 48°24’39.50”N, 24°24’50.28”E; **Identification:** identifiedBy: A. Nuzhna; dateIdentified: 2013; **Event:** samplingProtocol: sweeping; eventDate: 07/12/2004
- **Type status:** Other material. **Occurrence:** recordedBy: O. Varga; individualCount: 1; sex: female; lifeStage: adult; **Location:** country: Ukraine; stateProvince: Transcarpathian region; county: Rakhiv district; locality: Svydovets, subalpine zone, 8 km NW of Kvasy; verbatimElevation: 1600-1650 m; verbatimCoordinates: 48°12'39.16"N, 24°14'30.75"E; **Identification:** identifiedBy: A. Nuzhna; dateIdentified: 2013; **Event:** samplingProtocol: sweeping; eventDate: 09/27/2011
- **Type status:** Other material. **Occurrence:** recordedBy: O. Varga; individualCount: 2; sex: males; lifeStage: adult; **Location:** country: Ukraine; stateProvince: Transcarpathian region; county: Rakhiv district; locality: Marmarosy, ​12 km SE of Dilove; verbatimElevation: 1400-1500 m; verbatimCoordinates: 47°54'56.55"N, 24°17'43.41"E; **Identification:** identifiedBy: A. Nuzhna; dateIdentified: 2013; **Event:** samplingProtocol: sweeping; eventDate: 6-9.08.2011

#### Distribution

Holarctic and Oriental regions (Yu et al. 2012). Ukraine: Kyiv, Odesa, Volyn, Zhytomyr regions, and Crimea (Nuzhna 2013), Ivano-Frankivsk and Transcarpathian regions.

### Agrypon gracilipes

(Curtis, 1839)

#### Materials

- **Type status:** Other material. **Occurrence:** recordedBy: O. Varga; individualCount: 1; sex: female; lifeStage: adult; **Location:** country: Ukraine; stateProvince: Ivano-Frankivsk region; county: Bogorodchany district; locality: Mochary, mixed forest, 5 km NE of Bogorodchany; verbatimElevation: 300-350 m; verbatimCoordinates: 48°50'51.17"N, 24°35'26.91"E; **Identification:** identifiedBy: A. Nuzhna; dateIdentified: 2013; **Event:** samplingProtocol: sweeping; eventDate: 09/23/2010
- **Type status:** Other material. **Occurrence:** recordedBy: O. Varga; individualCount: 1; sex: female; lifeStage: adult; **Location:** country: Ukraine; stateProvince: Transcarpathian region; county: Rakhiv district; locality: Chornogora, beech forest, 4 km NE of Kvasy; verbatimElevation: 1000 m; verbatimCoordinates: 48°10'19.08"N, 24°18'09.16"E; **Identification:** identifiedBy: A. Nuzhna; dateIdentified: 2013; **Event:** samplingProtocol: sweeping; eventDate: 05/27/2011
- **Type status:** Other material. **Occurrence:** recordedBy: O. Varga; individualCount: 1; sex: male; lifeStage: adult; **Location:** country: Ukraine; stateProvince: Transcarpathian region; county: Rakhiv district; locality: Marmarosy, ​12 km SE of Dilove; verbatimElevation: 1400-1500 m; verbatimCoordinates: 47°54'56.55"N, 24°17'43.41"E; **Identification:** identifiedBy: A. Nuzhna; dateIdentified: 2013; **Event:** samplingProtocol: sweeping; eventDate: 07/17/1963
- **Type status:** Other material. **Occurrence:** recordedBy: M. Boganych; individualCount: 1; sex: male; lifeStage: adult; **Location:** country: Ukraine; stateProvince: Transcarpathian region; county: Khust district; locality: Khust; **Identification:** identifiedBy: A. Nuzhna; dateIdentified: 2013; **Event:** samplingProtocol: sweeping; eventDate: 6-9.08.2011
- **Type status:** Other material. **Occurrence:** recordedBy: O. Varga; individualCount: 3; sex: females; lifeStage: adult; **Location:** country: Ukraine; stateProvince: Transcarpathian region; county: Rakhiv district; locality: Marmarosy, ​12 km SE of Dilove; verbatimElevation: 1400-1500 m; verbatimCoordinates: 47°54'56.55"N, 24°17'43.41"E; **Identification:** identifiedBy: A. Nuzhna; dateIdentified: 2013; **Event:** samplingProtocol: sweeping; eventDate: 07/17/1963
- **Type status:** Other material. **Occurrence:** recordedBy: M. Boganych; individualCount: 3; sex: females; lifeStage: adult; **Location:** country: Ukraine; stateProvince: Transcarpathian region; county: Khust district; locality: Khust; **Identification:** identifiedBy: A. Nuzhna; dateIdentified: 2013; **Event:** samplingProtocol: sweeping; eventDate: 6-9.08.2011

#### Distribution

Palaearctic region (Yu et al. 2012). Ukraine: Kharkiv, Kherson, Kyiv, Odesa, Zaporizhzhya regions (Nuzhna 2013​)​, Ivano-Frankivsk and Transcarpathian regions.

### Agrypon flaveolatum

(Gravenhorst, 1807)

#### Materials

- **Type status:** Other material. **Occurrence:** recordedBy: O. Varga; individualCount: 1; sex: female; lifeStage: adult; **Location:** country: Ukraine; stateProvince: Transcarpathian region; county: Rakhiv district; locality: Marmarosy, ​12 km SE of Dilove; verbatimElevation: 1400-1500 m; verbatimCoordinates: 47°54'56.55"N, 24°17'43.41"E; **Identification:** identifiedBy: A. Nuzhna; dateIdentified: 2013; **Event:** samplingProtocol: sweeping; eventDate: 6-9.08.2011
- **Type status:** Other material. **Occurrence:** recordedBy: V. Tolkanitz; individualCount: 1; sex: female; lifeStage: adult; **Location:** country: Ukraine; stateProvince: Ternopil region; county: Borshchiv district; locality: Babintsy; verbatimElevation: 200 m; **Identification:** identifiedBy: A. Nuzhna; dateIdentified: 2013; **Event:** samplingProtocol: sweeping; eventDate: 06/01/1969

#### Distribution

Holarctic region (Yu et al. 2012). Ukraine: all regions (Nuzhna 2013).

### Agrypon flexorium

(Thunberg, 1824)

#### Materials

- **Type status:** Other material. **Occurrence:** recordedBy: O. Varga; individualCount: 1; sex: female; lifeStage: adult; **Location:** country: Ukraine; stateProvince: Ivano-Frankivsk region; county: Bogorodchany district; locality: Gorgany, coniferous forest, 11-12 km SW of Stara Guta; verbatimElevation: 1250-1300 m; verbatimCoordinates: 48°33’32.30”N, 24°07’41.34”E; **Identification:** identifiedBy: A. Nuzhna; dateIdentified: 2013; **Event:** samplingProtocol: sweeping; eventDate: 20-22.05.2012
- **Type status:** Other material. **Occurrence:** recordedBy: V. Tolkanitz; individualCount: 1; sex: female; lifeStage: adult; **Location:** country: Ukraine; stateProvince: Transcarpathian region; county: Svalyava district; verbatimElevation: 250 m; **Identification:** identifiedBy: A. Nuzhna; dateIdentified: 2013; **Event:** samplingProtocol: sweeping; eventDate: 07/09/1986
- **Type status:** Other material. **Occurrence:** recordedBy: O. Varga; individualCount: 1; sex: female; lifeStage: adult; **Location:** country: Ukraine; stateProvince: Transcarpathian region; county: Rakhiv district; locality: Sheshul, 8-9 km NE of Kvasy; verbatimElevation: 1600-1700 m; verbatimCoordinates: 48°09’23.13”N, 24°21’27.15”E; **Identification:** identifiedBy: A. Nuzhna; dateIdentified: 2013; **Event:** samplingProtocol: sweeping; eventDate: 06/17/2012
- **Type status:** Other material. **Occurrence:** recordedBy: O. Varga; individualCount: 3; sex: males; lifeStage: adult; **Location:** country: Ukraine; stateProvince: Transcarpathian region; county: Rakhiv district; locality: Marmarosy, ​12 km SE of Dilove; verbatimElevation: 1400-1500 m; verbatimCoordinates: 47°54'56.55"N, 24°17'43.41"E; **Identification:** identifiedBy: A. Nuzhna; dateIdentified: 2013; **Event:** samplingProtocol: sweeping; eventDate: 6-9.08.2011
- **Type status:** Other material. **Occurrence:** recordedBy: O. Varga; individualCount: 2; sex: females; lifeStage: adult; **Location:** country: Ukraine; stateProvince: Transcarpathian region; county: Rakhiv district; locality: Marmarosy, ​12 km SE of Dilove; verbatimElevation: 1400-1500 m; verbatimCoordinates: 47°54'56.55"N, 24°17'43.41"E; **Identification:** identifiedBy: A. Nuzhna; dateIdentified: 2013; **Event:** samplingProtocol: sweeping; eventDate: 6-9.08.2011

#### Distribution

**Figure 3.**
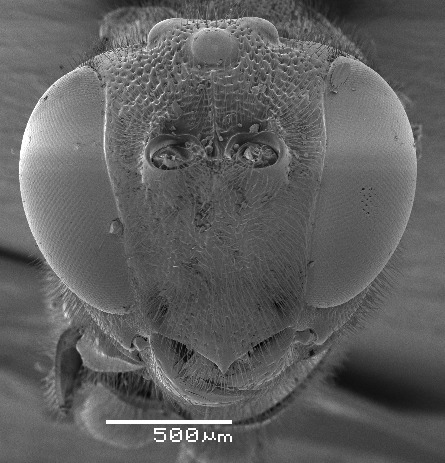
*Agrypon flexorium* (Thunberg, 1822), female, dorsal view of face.

Palaearctic region (Yu et al. 2012). Ukraine: all regions (Nuzhna 2013​) (Fig. 3).

### Agrypon flexorioides

Schnee, 1989

#### Materials

- **Type status:** Other material. **Occurrence:** recordedBy: A. Kotenko; individualCount: 1; sex: male; lifeStage: adult; **Location:** country: Ukraine; stateProvince: Transcarpathian region; county: Volovets district; locality: Pidpolozzya; verbatimElevation: 400 m; **Identification:** identifiedBy: A. Nuzhna; dateIdentified: 2013; **Event:** samplingProtocol: sweeping; eventDate: 07/14/1954
- **Type status:** Other material. **Occurrence:** recordedBy: O. Varga; individualCount: 1; sex: male; lifeStage: adult; **Location:** country: Ukraine; stateProvince: Ivano-Frankivsk region; county: Nadvirna district; locality: Gorgany, Elmy, coniferous forest, 15 km SW of Yaremche; verbatimElevation: 800-900 m; verbatimCoordinates: 48°24’39.50”N, 24°24’50.28”E; **Identification:** identifiedBy: A. Nuzhna; dateIdentified: 2013; **Event:** samplingProtocol: sweeping; eventDate: 07/17/2009

#### Distribution

Weatern Palaearctic region (Yu et al. 2012). Ukraine: Donetsk, Lugansk regions, and Crimea (Nuzhna 2013​), Ivano-Frankivsk and Transcarpathian regions.

### Agrypon interstitiale

Schnee, 1989

#### Materials

- **Type status:** Other material. **Occurrence:** recordedBy: O. Varga; individualCount: 1; sex: male; lifeStage: adult; **Location:** country: Ukraine; stateProvince: Transcarpathian region; county: Rakhiv district; locality: Chornogora, beech forest, 5 km NE of Kvasy; verbatimElevation: 1200 m; verbatimCoordinates: 48°10'02.78"N, 24°19'21.69"E; **Identification:** identifiedBy: A. Nuzhna; dateIdentified: 2013; **Event:** samplingProtocol: sweeping; eventDate: 06/15/2012
- **Type status:** Other material. **Occurrence:** recordedBy: O. Varga; individualCount: 1; sex: male; lifeStage: adult; **Location:** country: Ukraine; stateProvince: Transcarpathian region; county: Rakhiv district; locality: Marmarosy, ​12 km SE of Dilove; verbatimElevation: 1400-1500 m; verbatimCoordinates: 47°54'56.55"N, 24°17'43.41"E; **Identification:** identifiedBy: A. Nuzhna; dateIdentified: 2013; **Event:** samplingProtocol: sweeping; eventDate: 6-9.08.2011

#### Distribution

Western Palaearctic region (Yu et al. 2012). Ukraine: Donetsk and Kyiv regions, Crimea (Nuzhna 2013​), Transcarpathian region.

### Agrypon scutellatum

(Hellén, 1926)

#### Materials

- **Type status:** Other material. **Occurrence:** recordedBy: O. Varga; individualCount: 1; sex: male; lifeStage: adult; **Location:** country: Ukraine; stateProvince: Ivano-Frankisk region; county: Nadvirna district; locality: Chornogora, subalpine zone, slope of m. Goverla; verbatimElevation: 1400-1500 m; verbatimCoordinates: 48°10'10.89"N, 24°29'42.89"E; **Identification:** identifiedBy: A. Nuzhna; dateIdentified: 2013; **Event:** samplingProtocol: sweeping; eventDate: 07/28/2012
- **Type status:** Other material. **Occurrence:** recordedBy: O. Varga; individualCount: 1; sex: male; lifeStage: adult; **Location:** country: Ukraine; stateProvince: Transcarpathian region; county: Rakhiv district; locality: Marmarosy, ​12 km SE of Dilove; verbatimElevation: 1400-1500 m; verbatimCoordinates: 47°54'56.55"N, 24°17'43.41"E; **Identification:** identifiedBy: A. Nuzhna; dateIdentified: 2013; **Event:** samplingProtocol: sweeping; eventDate: 6-9.08.2011

#### Distribution

**Figure 5.**
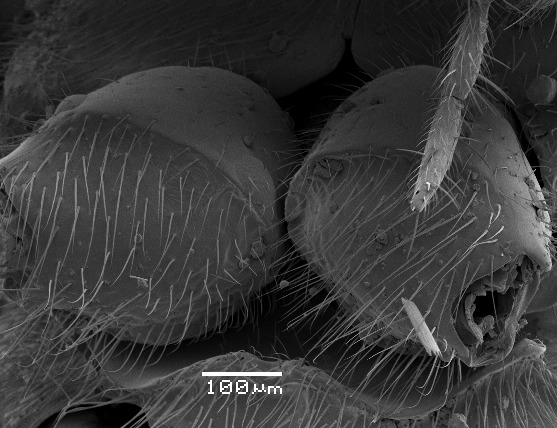
*Agrypon scutellatum* (Hellén, 1926), female, ventral view of fore coxae.

Europe, known only from Finland (Hellén 1950, Yu et al. 2012). Ukraine: Ivano-Frankivsk and Transcarpathian regions. New record for Ukraine (Fig. 4, Fig. 5).

### Agrypon varitarsum

(Wesmael, 1849)

#### Materials

- **Type status:** Other material. **Occurrence:** recordedBy: O. Varga; individualCount: 1; sex: male; lifeStage: adult; **Location:** country: Ukraine; stateProvince: Ivano-Frankisk region; county: Bogorodchany district; locality: Mochary, mixed forest, 5 km NE of Bogorodchany; verbatimElevation: 300-350 m; verbatimCoordinates: 48°50'51.17"N, 24°35'26.91"E; **Identification:** identifiedBy: A. Nuzhna; dateIdentified: 2013; **Event:** samplingProtocol: sweeping; eventDate: 09/23/2010
- **Type status:** Other material. **Occurrence:** recordedBy: O. Varga; individualCount: 2; sex: females; lifeStage: adult; **Location:** country: Ukraine; stateProvince: Ivano-Frankisk region; county: Bogorodchany district; locality: Mochary, mixed forest, 5 km NE of Bogorodchany; verbatimElevation: 300-350 m; verbatimCoordinates: 48°50'51.17"N, 24°35'26.91"E; **Identification:** identifiedBy: A. Nuzhna; dateIdentified: 2013; **Event:** samplingProtocol: sweeping; eventDate: 09/23/2010
- **Type status:** Other material. **Occurrence:** recordedBy: O. Varga; individualCount: 1; sex: female; lifeStage: adult; **Location:** country: Ukraine; stateProvince: Ivano-Frankisk region; county: Bogorodchany district; locality: Mochary, mixed forest, 5 km NE of Bogorodchany; verbatimElevation: 300-350 m; verbatimCoordinates: 48°50'51.17"N, 24°35'26.91"E; **Identification:** identifiedBy: A. Nuzhna; dateIdentified: 2013; **Event:** samplingProtocol: sweeping; eventDate: 09/23/2010
- **Type status:** Other material. **Occurrence:** recordedBy: O. Varga; individualCount: 1; sex: female; lifeStage: adult; **Location:** country: Ukraine; stateProvince: Transcarpathian region; county: Rakhiv district; locality: Marmarosy, ​12 km SE of Dilove; verbatimElevation: 1400-1500 m; verbatimCoordinates: 47°54'56.55"N, 24°17'43.41"E; **Identification:** identifiedBy: A. Nuzhna; dateIdentified: 2013; **Event:** samplingProtocol: sweeping; eventDate: 6-9.08.2011
- **Type status:** Other material. **Occurrence:** recordedBy: O. Varga; individualCount: 4; sex: females; lifeStage: adult; **Location:** country: Ukraine; stateProvince: Transcarpathian region; county: Rakhiv district; locality: Svydovets, beech forest, 2-3 km NW of Kvasy; verbatimElevation: 850-900 m; verbatimCoordinates: 48°09'08.89"N, 24°15'58.35"E; **Identification:** identifiedBy: A. Nuzhna; dateIdentified: 2013; **Event:** samplingProtocol: Malaise trap; eventDate: 14.07-24.08.2013

#### Distribution

Holarctic and Oriental regions (Yu et al. 2012). Ukraine: Kheson and Kyiv regions, Crimea (Nuzhna 2013​)​, Ivano-Frankivsk and Transcarpathian regions.

### Aphanistes gliscens

(Hartig, 1838)

#### Materials

- **Type status:** Other material. **Occurrence:** recordedBy: A. Sirenko; individualCount: 1; sex: female; lifeStage: adult; **Location:** country: Ukraine; stateProvince: Ivano-Frankivsk region; county: Rogatyn district; locality: Vyshniv; verbatimElevation: 300 m; **Identification:** identifiedBy: A. Nuzhna; dateIdentified: 2013; **Event:** samplingProtocol: sweeping; eventDate: 08/01/2006
- **Type status:** Other material. **Occurrence:** recordedBy: O. Varga; individualCount: 1; sex: female; lifeStage: adult; **Location:** country: Ukraine; stateProvince: Transcarpathian region; county: Rakhiv district; locality: Marmarosy, 12 km SE of Dilove; verbatimElevation: 1400-1500 m; verbatimCoordinates: 47°54'56.55"N, 24°17'43.41"E; **Identification:** identifiedBy: A. Nuzhna; dateIdentified: 2013; **Event:** samplingProtocol: sweeping; eventDate: 6-9.08.2011

#### Distribution

Palaearctic region (Yu et al. 2012). Ukraine: Dnipropetrovsk, Kherson, Kyiv, Rivne Regions (Nuzhna, unpubl.), Ivano-Frankivsk and Transcarpathian regions. New record for Ukraine.

### Aphanistes klugii

(Hartig, 1838)

#### Materials

- **Type status:** Other material. **Occurrence:** recordedBy: O. Varga; individualCount: 2; sex: males; lifeStage: adult; **Location:** country: Ukraine; stateProvince: Transcarpathian region; county: Vynogradiv district; locality: Chorna Gora; verbatimElevation: 300 m; verbatimCoordinates: 48°09'19.70"N, 23°04'22.47"E; **Identification:** identifiedBy: A. Nuzhna; dateIdentified: 2013; **Event:** samplingProtocol: sweeping; eventDate: 04/06/2011
- **Type status:** Other material. **Occurrence:** recordedBy: O. Varga; individualCount: 1; sex: female; lifeStage: adult; **Location:** country: Ukraine; stateProvince: Transcarpathian region; county: Rakhiv district; locality: Marmarosy, 12 km SE of Dilove; verbatimElevation: 1400-1500 m; verbatimCoordinates: 47°54'56.55"N, 24°17'43.41"E; **Identification:** identifiedBy: A. Nuzhna; dateIdentified: 2013; **Event:** samplingProtocol: sweeping; eventDate: 6-9.08.2011

#### Distribution

Palaearctic region (Yu et al. 2012). Ukraine: Kharkiv and Kyiv regions (Nuzhna, unpubl.) Transcarpathian region. New record for Ukraine.

### Aphanistes ruficornis

(Gravenhorst, 1829)

#### Materials

- **Type status:** Other material. **Occurrence:** recordedBy: O. Varga; individualCount: 2; sex: females; lifeStage: adult; **Location:** country: Ukraine; stateProvince: Transcarpathian region; county: Rakhiv district; locality: Marmarosy, 12 km SE of Dilove; verbatimElevation: 1400-1500 m; verbatimCoordinates: 47°54'56.55"N, 24°17'43.41"E; **Identification:** identifiedBy: A. Nuzhna; dateIdentified: 2013; **Event:** samplingProtocol: sweeping; eventDate: 08/11/2009
- **Type status:** Other material. **Occurrence:** recordedBy: O. Varga; individualCount: 1; sex: male; lifeStage: adult; **Location:** country: Ukraine; stateProvince: Transcarpathian region; county: Rakhiv district; locality: Marmarosy, 12 km SE of Dilove; verbatimElevation: 1400-1500 m; verbatimCoordinates: 47°54'56.55"N, 24°17'43.41"E; **Identification:** identifiedBy: A. Nuzhna; dateIdentified: 2013; **Event:** samplingProtocol: sweeping; eventDate: 6-9.08.2011

#### Distribution

Palaearctic region (Yu et al. 2012). Ukraine: Donetsk, Kyiv, Vinnytsya regions and Crimea (Nuzhna 2010), Transcarpathian region.

### Barylypa delictor

(Thunberg, 1824)

#### Materials

- **Type status:** Other material. **Occurrence:** recordedBy: O. Varga; individualCount: 1; sex: female; lifeStage: adult; **Location:** country: Ukraine; stateProvince: Ternopil region; locality: Zalishchyky; verbatimElevation: 200 m; **Identification:** identifiedBy: A. Nuzhna; dateIdentified: 2013; **Event:** samplingProtocol: sweeping; eventDate: 05/12/2011
- **Type status:** Other material. **Occurrence:** recordedBy: V. Tolkanitz; individualCount: 1; sex: female; lifeStage: adult; **Location:** country: Ukraine; stateProvince: Ternopil region; county: Borshchiv district; locality: Babintsy; verbatimElevation: 200 m; **Identification:** identifiedBy: A. Nuzhna; dateIdentified: 2013; **Event:** samplingProtocol: sweeping; eventDate: 06/01/1969
- **Type status:** Other material. **Occurrence:** recordedBy: O. Varga; individualCount: 1; sex: female; lifeStage: adult; **Location:** country: Ukraine; stateProvince: Transcarpathian region; county: Rakhiv district; locality: Svydovets, beech forest, 2-3 km NW of Kvasy; verbatimElevation: 850-900 m; verbatimCoordinates: 48°09'08.89"N, 24°15'58.35"E; **Identification:** identifiedBy: A. Nuzhna; dateIdentified: 2013; **Event:** samplingProtocol: Malaise trap; eventDate: 14.07-24.08.2013

#### Distribution

**Figure 6.**
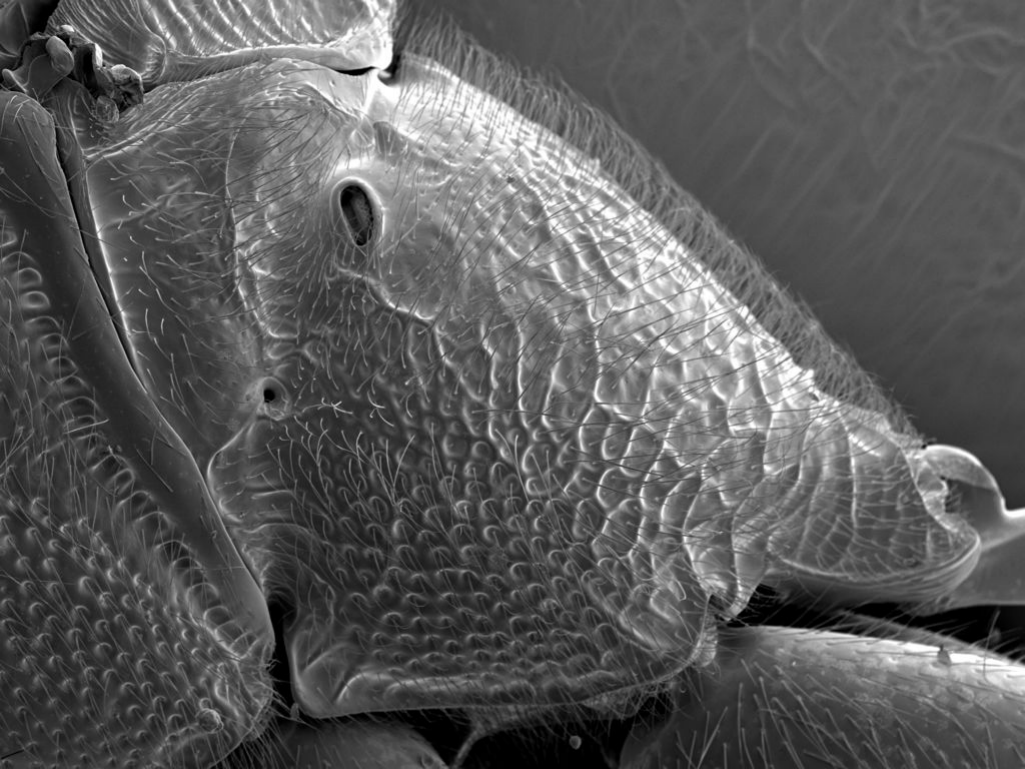
*Barylypa delictor* (Thunberg, 1822), female, lateral view of propodeum.

Palaearctic region (Yu et al. 2012). Ukraine: all regions (Atanasov 1981) (Fig. 6).

### Heteropelma amictum

(Fabricius, 1775)

#### Materials

- **Type status:** Other material. **Occurrence:** recordedBy: O. Varga; individualCount: 1; sex: female; lifeStage: adult; **Location:** country: Ukraine; stateProvince: Ivano-Frankivsk region; county: Bogorodchany district; locality: Mochary, mixed forest, 5 km NE to Bogorodchany; verbatimElevation: 300-350 m; verbatimCoordinates: 48°50'51.17"N, 24°35'26.91"E; **Identification:** identifiedBy: A. Nuzhna; dateIdentified: 2013; **Event:** samplingProtocol: sweeping; eventDate: 08/15/2011
- **Type status:** Other material. **Occurrence:** recordedBy: O. Varga; individualCount: 1; sex: female; lifeStage: adult; **Location:** country: Ukraine; stateProvince: Ivano-Frankivsk region; county: Bogorodchany district; locality: Gorgany, coniferous forest, 10-12 km of Stara Guta; verbatimElevation: 1200-1300 m; verbatimCoordinates: 48°33'7.45"N, 24°11'55.33"E; **Identification:** identifiedBy: A. Nuzhna; dateIdentified: 2013; **Event:** samplingProtocol: sweeping; eventDate: 2011-08-17/19
- **Type status:** Other material. **Occurrence:** recordedBy: O. Varga; individualCount: 1; sex: male; lifeStage: adult; **Location:** country: Ukraine; stateProvince: Ivano-Frankivsk region; county: Yaremche district; locality: Yablunytsya, coniferous forest; **Identification:** identifiedBy: A. Nuzhna; dateIdentified: 2013; **Event:** samplingProtocol: sweeping; eventDate: 07/11/2006
- **Type status:** Other material. **Occurrence:** recordedBy: V Ermolenko; individualCount: 1; sex: male; lifeStage: adult; **Location:** country: Ukraine; stateProvince: Lviv region; locality: Skole; verbatimElevation: 450-500 m; **Identification:** identifiedBy: A. Nuzhna; dateIdentified: 2013; **Event:** samplingProtocol: sweeping; eventDate: 07/13/1976
- **Type status:** Other material. **Occurrence:** recordedBy: V. Ermolenko; individualCount: 2; sex: females; lifeStage: adult; **Location:** country: Ukraine; stateProvince: Lviv region; locality: Skole; verbatimElevation: 450-500 m; **Identification:** identifiedBy: A. Nuzhna; dateIdentified: 2013; **Event:** samplingProtocol: sweeping; eventDate: 07/13/1976
- **Type status:** Other material. **Occurrence:** recordedBy: V. Ermolenko; individualCount: 1; sex: female; lifeStage: adult; **Location:** country: Ukraine; stateProvince: Transcarpathian region; county: Rakhiv district; locality: Rakhiv, beech forest; verbatimElevation: 450-500 m; **Identification:** identifiedBy: A. Nuzhna; dateIdentified: 2013; **Event:** samplingProtocol: sweeping; eventDate: 08/12/1961
- **Type status:** Other material. **Occurrence:** recordedBy: O. Varga; individualCount: 1; sex: male; lifeStage: adult; **Location:** country: Ukraine; stateProvince: Transcarpathian region; county: Rakhiv district; locality: Svydovets, beech forest, 4 km NW of Kvasy; verbatimElevation: 1000 m; verbatimCoordinates: 48°10'19.08"N, 24°18'09.16"E; **Identification:** identifiedBy: A. Nuzhna; dateIdentified: 2013; **Event:** samplingProtocol: sweeping; eventDate: 08/16/2009
- **Type status:** Other material. **Occurrence:** recordedBy: A. Kotenko; individualCount: 1; sex: female; lifeStage: adult; **Location:** country: Ukraine; stateProvince: Transcarpathian region; county: Tyachiv district; locality: Mala Ugolka, beech forest; verbatimElevation: 600-700 m; **Identification:** identifiedBy: A. Nuzhna; dateIdentified: 2013; **Event:** samplingProtocol: sweeping; eventDate: 07/27/1975

#### Distribution

Australasian, Palaearctic, and Oriental regions (Yu et al. 2012). Ukraine: Ivano-Frankivsk, Lviv, and Transcarpathian regions. New record for Ukraine (Fig. 7).

### Heteropelma megarthrum

(Ratzeburg, 1848)

#### Materials

- **Type status:** Other material. **Occurrence:** recordedBy: O. Varga; individualCount: 1; sex: female; lifeStage: adult; **Location:** country: Ukraine; stateProvince: Ivano-Frankivsk region; county: Bogorodchany district; locality: Mochary, mixed forest, 5 km NE of Bogorodchany; verbatimElevation: 300-350 m; verbatimCoordinates: 48°50'51.17"N, 24°35'26.91"E; **Identification:** identifiedBy: A. Nuzhna; dateIdentified: 2013; **Event:** samplingProtocol: sweeping; eventDate: 08/15/2011
- **Type status:** Other material. **Occurrence:** recordedBy: O. Varga; individualCount: 1; sex: female; lifeStage: adult; **Location:** country: Ukraine; stateProvince: Ivano-Frankivsk region; county: Bogorodchany district; locality: Gorgany, coniferous forest, 10-12 km of Stara Guta; verbatimElevation: 1200-1300 m; verbatimCoordinates: 48°33'7.45"N, 24°11'55.33"E; **Identification:** identifiedBy: A. Nuzhna; dateIdentified: 2013; **Event:** samplingProtocol: sweeping; eventDate: 2011-08-17/19
- **Type status:** Other material. **Occurrence:** recordedBy: O. Varga; individualCount: 1; sex: male; lifeStage: adult; **Location:** country: Ukraine; stateProvince: Ivano-Frankivsk region; county: Bogorodchany district; locality: Gorgany, coniferous forest, 5 km SW of Stara Guta; verbatimElevation: 1200 m; verbatimCoordinates: 48°36'42.77"N, 24°09'10.69"E; **Identification:** identifiedBy: A. Nuzhna; dateIdentified: 2013; **Event:** samplingProtocol: sweeping; eventDate: 06/20/2013
- **Type status:** Other material. **Occurrence:** recordedBy: A. Kotenko; individualCount: 1; sex: male; lifeStage: adult; **Location:** country: Ukraine; stateProvince: Transcarpathian region; county: Rakhiv district; locality: Chornogora, coniferous forest; verbatimElevation: 800-1000 m; **Identification:** identifiedBy: A. Nuzhna; dateIdentified: 2013; **Event:** samplingProtocol: sweeping; eventDate: 08/05/1994
- **Type status:** Other material. **Occurrence:** recordedBy: V. Tolkanitz; individualCount: 2; sex: females; lifeStage: adult; **Location:** country: Ukraine; stateProvince: Transcarpathian region; county: Svalyava district; locality: Polyana; verbatimElevation: 250 m; **Identification:** identifiedBy: A. Nuzhna; dateIdentified: 2013; **Event:** samplingProtocol: sweeping; eventDate: 06/26/1986

#### Distribution

Palaearctic and Oriental regions (Yu et al. 2012). Ukraine: all regions (Nuzhna 2010).

### Perisphincter gracilicornis

Schnee, 1978

#### Materials

- **Type status:** Other material. **Occurrence:** recordedBy: O. Varga; individualCount: 1; sex: male; lifeStage: adult; **Location:** country: Ukraine; stateProvince: Ivano-Frankivsk region; county: Bogorodchany district; locality: Mochary, mixed forest, 5 km NE of Bogorodchany; verbatimElevation: 300-350 m; verbatimCoordinates: 48°50'51.17"N, 24°35'26.91"E; **Identification:** identifiedBy: A. Nuzhna; dateIdentified: 2013; **Event:** samplingProtocol: sweeping; eventDate: 06/04/2011
- **Type status:** Other material. **Occurrence:** recordedBy: O. Varga; individualCount: 1; sex: female; lifeStage: adult; **Location:** country: Ukraine; stateProvince: Transcarpathian region; county: Rakhiv district; locality: Svydovets, beech forest, 2-3 km NW of Kvasy; verbatimElevation: 850-900 m; verbatimCoordinates: 48°09'08.89"N, 24°15'58.35"E; **Identification:** identifiedBy: A. Nuzhna; dateIdentified: 2013; **Event:** samplingProtocol: Malaise trap; eventDate: 07/14/2013

#### Distribution

Europe, known only from Germany and Poland (Yu et al. 2012). Ukraine: Kiyv region (Nuzhna, unpubl.), Ivano-Frankivsk and Transcarpathian regions. New record for Ukraine.

### Therion circumflexum

(Linnaeus, 1758)

#### Materials

- **Type status:** Other material. **Occurrence:** recordedBy: O. Varga; individualCount: 1; sex: female; lifeStage: adult; **Location:** country: Ukraine; stateProvince: Ivano-Frankivsk region; locality: Gorgany, coniferous forest, 8-10 km E of Vorokhta; verbatimElevation: 900-1000 m; verbatimCoordinates: 48°14’02.91”N, 24°39’14.68”E; **Identification:** identifiedBy: A. Nuzhna; dateIdentified: 2013; **Event:** samplingProtocol: sweeping; eventDate: 08/01/2010
- **Type status:** Other material. **Occurrence:** recordedBy: O. Varga; individualCount: 1; sex: male; lifeStage: adult; **Location:** country: Ukraine; stateProvince: Ivano-Frankivsk region; county: Bogorodchany district; locality: Zhbyr, mixed forest, 7-8 km SW of Bogorodchany; verbatimElevation: 400 m; verbatimCoordinates: 48°47'4.92"N, 24°28'46.45"E; **Identification:** identifiedBy: A. Nuzhna; dateIdentified: 2013; **Event:** samplingProtocol: sweeping; eventDate: 09/20/2011
- **Type status:** Other material. **Occurrence:** recordedBy: O. Varga; individualCount: 1; sex: male; lifeStage: adult; **Location:** country: Ukraine; stateProvince: Ivano-Frankivsk region; county: Bogorodchany district; locality: Zhbyr, mixed forest, 7-8 km SW of Bogorodchany; verbatimElevation: 400 m; verbatimCoordinates: 48°47'4.92"N, 24°28'46.45"E; **Identification:** identifiedBy: A. Nuzhna; dateIdentified: 2013; **Event:** samplingProtocol: sweeping; eventDate: 09/20/2011
- **Type status:** Other material. **Occurrence:** recordedBy: O. Varga; individualCount: 1; sex: female; lifeStage: adult; **Location:** country: Ukraine; stateProvince: Ivano-Frankivsk region; county: Bogorodchany district; locality: Mochary, mixed forest, 5 km NE of Bogorodchany; verbatimElevation: 300-350 m; verbatimCoordinates: 48°50'51.17"N, 24°35'26.91"E; **Identification:** identifiedBy: A. Nuzhna; dateIdentified: 2013; **Event:** samplingProtocol: sweeping; eventDate: 09/03/2012
- **Type status:** Other material. **Occurrence:** recordedBy: O. Varga; individualCount: 3; sex: males; lifeStage: adult; **Location:** country: Ukraine; stateProvince: Ivano-Frankivsk region; county: Bogorodchany district; locality: Gorgany, pol. Playek, coniferous forest, 5 km SW of Stara Guta; verbatimElevation: 1200 m; verbatimCoordinates: 48°36'42.77"N, 24°09'10.69"E; **Identification:** identifiedBy: A. Nuzhna; dateIdentified: 2013; **Event:** samplingProtocol: sweeping; eventDate: 07/01/2012
- **Type status:** Other material. **Occurrence:** recordedBy: O. Varga; individualCount: 2; sex: males; lifeStage: adult; **Location:** country: Ukraine; stateProvince: Ivano-Frankivsk region; county: Bogorodchany district; locality: Gorgany, coniferous forest, 11-12 km of Stara Guta; verbatimElevation: 1200-1300 m; verbatimCoordinates: 48°33'7.45"N, 24°11'55.33"E; **Identification:** identifiedBy: A. Nuzhna; dateIdentified: 2013; **Event:** samplingProtocol: sweeping; eventDate: 2011-08-17/19
- **Type status:** Other material. **Occurrence:** recordedBy: O. Varga; individualCount: 1; sex: female; lifeStage: adult; **Location:** country: Ukraine; stateProvince: Ivano-Frankivsk region; county: Bogorodchany district; locality: Gorgany, coniferous forest, 11-12 km of Stara Guta; verbatimElevation: 1200-1300 m; verbatimCoordinates: 48°33'7.45"N, 24°11'55.33"E; **Identification:** identifiedBy: A. Nuzhna; dateIdentified: 2013; **Event:** samplingProtocol: sweeping; eventDate: 07/04/2010
- **Type status:** Other material. **Occurrence:** recordedBy: O. Varga; individualCount: 1; sex: male; lifeStage: adult; **Location:** country: Ukraine; stateProvince: Ivano-Frankivsk region; county: Bogorodchany district; locality: Gorgany, coniferous forest, 11-12 km of Stara Guta; verbatimElevation: 1200-1300 m; verbatimCoordinates: 48°33'7.45"N, 24°11'55.33"E; **Identification:** identifiedBy: A. Nuzhna; dateIdentified: 2013; **Event:** samplingProtocol: sweeping; eventDate: 06/04/2012
- **Type status:** Other material. **Occurrence:** recordedBy: O. Varga; individualCount: 3; sex: males; lifeStage: adult; **Location:** country: Ukraine; stateProvince: Ivano-Frankivsk region; county: Nadvirna district; locality: Gorgany, Elmy, coniferous forest, 15 km SW to Yaremche; verbatimElevation: 800-900 m; verbatimCoordinates: 48°24’39.50”N, 24°24’50.28”E; **Identification:** identifiedBy: A. Nuzhna; dateIdentified: 2013; **Event:** samplingProtocol: sweeping; eventDate: 2012-07-20/23
- **Type status:** Other material. **Occurrence:** recordedBy: O. Varga; individualCount: 1; sex: male; lifeStage: adult; **Location:** country: Ukraine; stateProvince: Transcarpathian region; county: Rakhiv district; locality: Svydovets, subalpine zone, 4 km NW of Kvasy; verbatimElevation: 1200-1300 m; verbatimCoordinates: 48°12'39.16"N, 24°14'30.75"E; **Identification:** identifiedBy: A. Nuzhna; dateIdentified: 2013; **Event:** samplingProtocol: sweeping; eventDate: 05/15/2012
- **Type status:** Other material. **Occurrence:** recordedBy: R. Bidychak; individualCount: 1; sex: female; lifeStage: adult; **Location:** country: Ukraine; stateProvince: Transcarpathian region; county: Tyachiv district; locality: Mala Ugolka, beech forest; verbatimElevation: 500-600 m; verbatimCoordinates: 48°10'02.27"N, 23°40'20.65"E; **Identification:** identifiedBy: A. Nuzhna; dateIdentified: 2013; **Event:** samplingProtocol: on light; eventDate: 05/16/2004

#### Distribution

Palaearctic and Oriental regions (Yu et al. 2012). Ukraine: all regions (Atanasov 1981).

### Therion giganteum

(Gravenhorst, 1829)

#### Materials

- **Type status:** Other material. **Occurrence:** recordedBy: O. Varga; individualCount: 1; sex: male; lifeStage: adult; **Location:** country: Ukraine; stateProvince: Ivano-Frankivsk region; county: Bogorodchany district; locality: Gorgany, coniferous forest, 10-12 km of Stara Guta; verbatimElevation: 1200-1300 m; verbatimCoordinates: 48°33'7.45"N, 24°11'55.33"E; **Identification:** identifiedBy: A. Nuzhna; dateIdentified: 2013; **Event:** samplingProtocol: sweeping; eventDate: 2011-08-17/19
- **Type status:** Other material. **Occurrence:** recordedBy: O. Varga; individualCount: 2; sex: males; lifeStage: adult; **Location:** country: Ukraine; stateProvince: Ivano-Frankivsk region; county: Nadvirna district; locality: Gorgany, Elmy, coniferous forest, 15 km SW to Yaremche; verbatimElevation: 800-900 m; verbatimCoordinates: 48°24’39.50”N, 24°24’50.28”E; **Identification:** identifiedBy: A. Nuzhna; dateIdentified: 2013; **Event:** samplingProtocol: sweeping; eventDate: 07/14/2011

#### Distribution

Palaearctic region (Yu et al. 2012). Ukraine: Donetsk, Kyiv, and Lugansk regions (Nuzhna, upubl.), Transcarpathian region. New record for Ukraine (Fig. 8).

### Trichomma enecator

(Rossi, 1790)

#### Materials

- **Type status:** Other material. **Occurrence:** recordedBy: O. Varga; individualCount: 3; sex: males; lifeStage: adult; **Location:** country: Ukraine; stateProvince: Ivano-Frankivsk region; county: Bogorodchany district; locality: Mochary, mixed forest, 5 km NE of Bogorodchany; verbatimElevation: 300-350 m; verbatimCoordinates: 48°50'51.17"N, 24°35'26.91"E; **Identification:** identifiedBy: A. Nuzhna; dateIdentified: 2013; **Event:** samplingProtocol: sweeping; eventDate: 08/31/2009
- **Type status:** Other material. **Occurrence:** recordedBy: O. Varga; individualCount: 1; sex: female; lifeStage: adult; **Location:** country: Ukraine; stateProvince: Ivano-Frankivsk region; county: Bogorodchany district; locality: Mochary, mixed forest, 5 km NE of Bogorodchany; verbatimElevation: 300-350 m; verbatimCoordinates: 48°50'51.17"N, 24°35'26.91"E; **Identification:** identifiedBy: A. Nuzhna; dateIdentified: 2013; **Event:** samplingProtocol: sweeping; eventDate: 07/07/2011
- **Type status:** Other material. **Occurrence:** recordedBy: O. Varga; individualCount: 1; sex: male; lifeStage: adult; **Location:** country: Ukraine; stateProvince: Ivano-Frankivsk region; county: Bogorodchany district; locality: Mochary, mixed forest, 5 km NE of Bogorodchany; verbatimElevation: 300-350 m; verbatimCoordinates: 48°50'51.17"N, 24°35'26.91"E; **Identification:** identifiedBy: A. Nuzhna; dateIdentified: 2013; **Event:** samplingProtocol: sweeping; eventDate: 09/03/2011
- **Type status:** Other material. **Occurrence:** recordedBy: O. Varga; individualCount: 1; sex: male; lifeStage: adult; **Location:** country: Ukraine; stateProvince: Ivano-Frankivsk region; county: Bogorodchany district; locality: Mochary, mixed forest, 5 km NE of Bogorodchany; verbatimElevation: 300-350 m; verbatimCoordinates: 48°50'51.17"N, 24°35'26.91"E; **Identification:** identifiedBy: A. Nuzhna; dateIdentified: 2013; **Event:** samplingProtocol: sweeping; eventDate: 04/20/2012
- **Type status:** Other material. **Occurrence:** recordedBy: A. Kotenko; individualCount: 1; sex: female; lifeStage: adult; **Location:** country: Ukraine; stateProvince: Transcarpathian region; county: Hust district; locality: Narcissus Valley; verbatimElevation: 200-250 m; verbatimCoordinates: 48°10'25.98"N, 23°22'17.19"E; **Identification:** identifiedBy: A. Nuzhna; dateIdentified: 2013; **Event:** samplingProtocol: sweeping; eventDate: 08/01/1995
- **Type status:** Other material. **Occurrence:** recordedBy: V. Tolkanitz; individualCount: 1; sex: female; lifeStage: adult; **Location:** country: Ukraine; stateProvince: Transcarpathian region; county: Rakhiv district; locality: Bogdan; verbatimElevation: 700-800 m; verbatimCoordinates: 48°02'02.65"N, 24°21'48.33"E; **Identification:** identifiedBy: A. Nuzhna; dateIdentified: 2013; **Event:** samplingProtocol: sweeping; eventDate: 07/28/1972

#### Distribution

**Figure 9.**
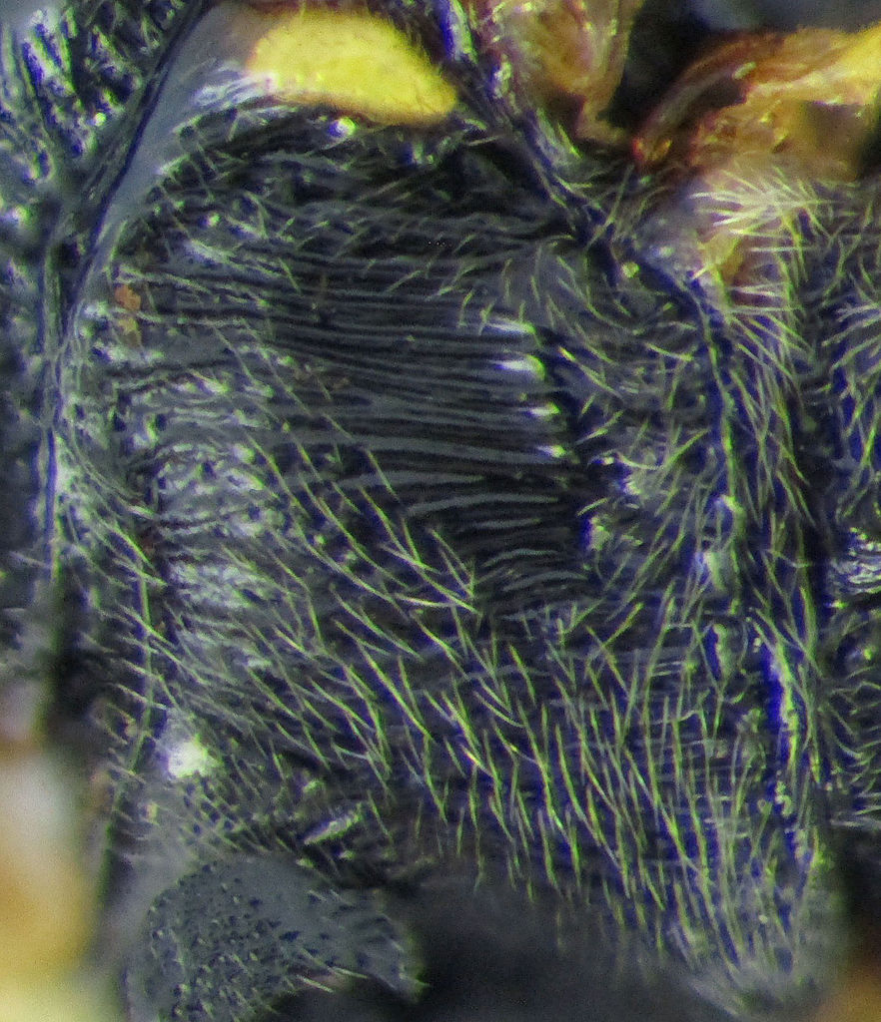
*Trichomma enecator* (Rossi, 1790), male, lateral view of mesopleuron.

Palaearctic region (Yu et al. 2012). Ukraine: all regions (Atanasov 1981) (Fig. 9).

### Trichomma fulvidens

Wesmael, 1849

#### Materials

- **Type status:** Other material. **Occurrence:** recordedBy: O. Varga; individualCount: 2; sex: males; lifeStage: adult; **Location:** country: Ukraine; stateProvince: Ivano-Frankivsk region; county: Bogorodchany district; locality: Mochary, mixed forest, 5 km NE of Bogorodchany; verbatimElevation: 300-350 m; verbatimCoordinates: 48°50'51.17"N, 24°35'26.91"E; **Identification:** identifiedBy: A. Nuzhna; dateIdentified: 2013; **Event:** samplingProtocol: sweeping; eventDate: 04/20/2012
- **Type status:** Other material. **Occurrence:** recordedBy: O. Varga; individualCount: 1; sex: female; lifeStage: adult; **Location:** country: Ukraine; stateProvince: Ivano-Frankivsk region; county: Bogorodchany district; locality: Mochary, mixed forest, 5 km NE of Bogorodchany; verbatimElevation: 300-350 m; verbatimCoordinates: 48°50'51.17"N, 24°35'26.91"E; **Identification:** identifiedBy: A. Nuzhna; dateIdentified: 2013; **Event:** samplingProtocol: sweeping; eventDate: 2012-05-18/19
- **Type status:** Other material. **Occurrence:** recordedBy: O. Varga; individualCount: 1; sex: male; lifeStage: adult; **Location:** country: Ukraine; stateProvince: Transcarpathian region; county: Vynohradiv district; locality: Chorna Gora; verbatimElevation: 500 m; verbatimCoordinates: 48°09'19.70"N, 23°04'22.47"E; **Identification:** identifiedBy: A. Nuzhna; dateIdentified: 2013; **Event:** samplingProtocol: sweeping; eventDate: 04/06/2011

#### Distribution

**Figure 10.**
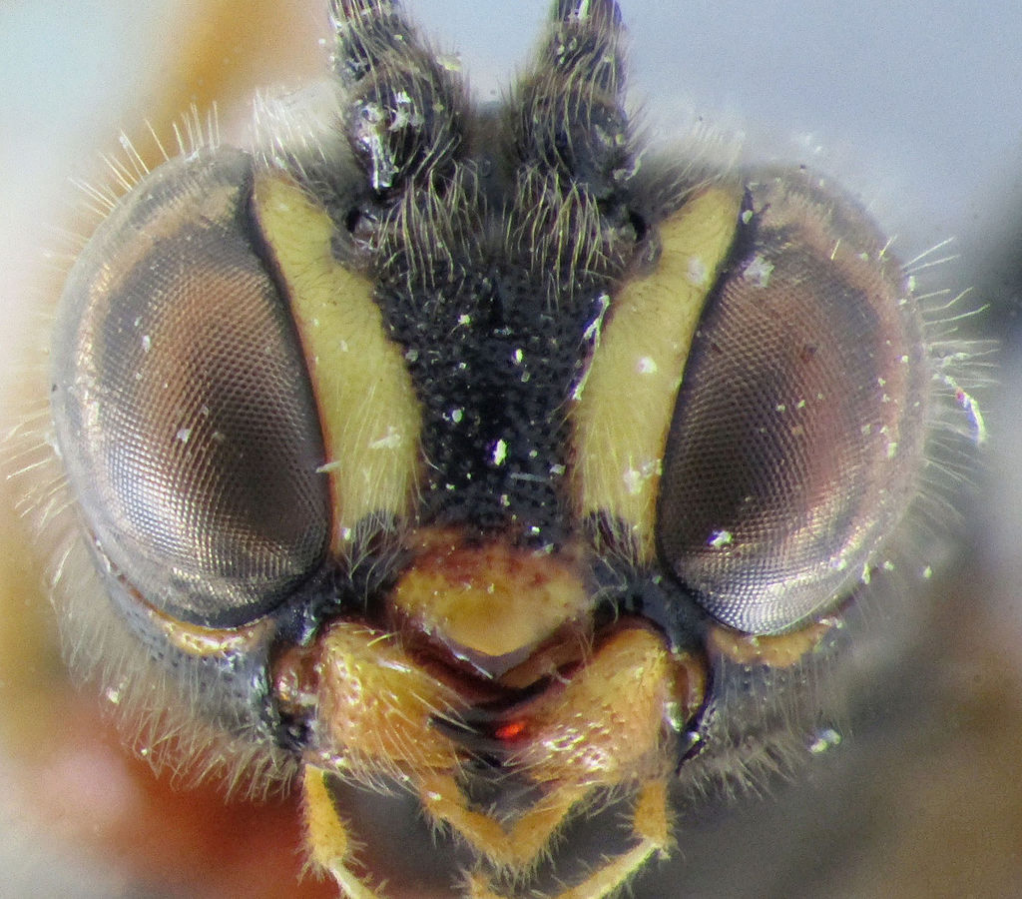
*Trichomma fulvidens* Wesmael, 1849, male, dorsal view of face.

Palaearctic region (Yu et al. 2012). Ukraine: Chernihiv, Donetsk, Kyiv, Lugansk, Odesa, and Poltava regions (Nuzhna, unpubl.), Ivano-Frankivsk and Transcarpathian regions. New record for Ukraine (Fig. 10).

### Trichomma occisor

Habermehl, 1909

#### Materials

- **Type status:** Other material. **Occurrence:** recordedBy: O. Varga; individualCount: 1; sex: female; lifeStage: adult; **Location:** country: Ukraine; stateProvince: Ivano-Frankivsk region; county: Bogorodchany district; locality: Mochary, mixed forest, 5 km NE of Bogorodchany; verbatimElevation: 300-350 m; verbatimCoordinates: 48°50'51.17"N, 24°35'26.91"E; **Identification:** identifiedBy: A. Nuzhna; dateIdentified: 2013; **Event:** samplingProtocol: sweeping; eventDate: 07/07/2011

#### Distribution

Palaearctic region (Yu et al. 2012). Ukraine: Ivano-Frankivsk region. New record for Ukraine (Fig. 11).

## Discussion

During investigations carried out in various locations of the Ukrainian Carpathians in 2009–2014 twenty four Anomaloninae species belonging to eight genera were recorded. Nine species, *Agrypon batis* Ratzeburg, 1955, *A. scutellatum* Hellén, 1926, *Aphnistes gliscens* Hartig, 1838, *A. klugii* Hartig, 1838, *Heteropelma amictum* Fabricius, 1775, *Perisphincter gracilicornis* Schnee, 1978, *Therion giganteum* Gravenhorst, 1829, *Trichomma fulvidens* Wesmael, 1849, *T. occisor* Habermehl, 1909 were recorded for the first time in Ukraine.

Anomaloninae species have been recorded in various high-altitude zones of the Ukrainian Carpathians (Table 1​). Anomaloninae species were the most abundant in the foothill oak forest zone, reaching up to 150–400 m a. s. l. in Precarpathia and Transcarpathian lowland with mixed forests, where the main tree species are *Quercus robur*, *Q. rubra*, *Caprinus* sp., *Fraxinus* sp., *Picea abies*, *Abies alba*, and *Pinus sylvestris*, where sixteen species were collected. Ten species were found in the beech forest zone (400–1300 m a. s. l.) in Transcarpathia. Eight species were found in the coniferous boreal forest zone, situated at 900–1600 m a. s. l. in the mountainous part of the Carpathians, where the *Piceeta-abietis* community predominates. Fourteen species, mainly *Agrypon* and *Aphanistes* species, and one *Therion* species (*Th. circumflexum*) were also collected in the subalpine zone, at 1400–2061 m a. s. l., known as polonynys, which are high altitude open grasslands, partly taken over by bush. Only the widespread and common *Th. circumflexum* was found in all high-altitude zones, six species were collected in three zones, nine species were found in two zones, and eight species – in one zone. It is notable that most the interesting rare species and new Ukrainian records were collected mainly from the lowest and highest altitudes: in the foothill oak forest zone and subalpine zone.

The flight season of Anomaloninae adults is prolonged, lasting six months, from the beginning of April to the end of September (Table 2​). The typical ‘steppe’ species, *Anomalon cruentatum*, was collected just at the end of June. This species in Central Ukraine (Kyiv region) has two emergences of adults: from the end of May to the middle of June and from the end of August to September, while in South Ukraine (Kherson region) it was collected from the end of June to the end of August. Two *Agrypon* species, *A. anomelas* and *A. flexorioides*, were collected at the beginning of August and in the middle of July, respectively. The remaining species of the genus have more prolonged flight periods lasting from one to almost three months usually late May – August. Only three species:, *A. clandestinum*, *A. gracilipes* and *A. varitarsum*, can be found in the Carpathians to the end of September. All three recorded *Aphanistes* species were collected during August, and in addition, *A. klugii* was found also at the beginning of April. *Barylypa delictor* was collected from May to August and *Heteropelma* species were found in the Carpathians from June to August. The flight period of *Perisphincter gracilicornis* in the Carpathians lasts from the beginning of June to the middle of July, while in Central Ukraine this species was collected from the middle of May untill the end of June. Probably the most common species, *Therion circumflexum*, was collected from May to September, another recorded species of the genus, *Th giganteum*, was found only in the middle of July and at the beginning of August. Among all Anomaloninae species *Trichomma enecator* has the longest flight period lasting from May to September, confirming the data from the other regions of Ukraine. The other two species, *T. fulvidens* and *T. occisor* were collected just during April and in the middle of June, respectively.

## Supplementary Material

XML Treatment for Anomalon cruentatum

XML Treatment for Agrypon anomelas

XML Treatment for Agrypon anxium

XML Treatment for Agrypon batis

XML Treatment for Agrypon clandestinum

XML Treatment for Agrypon gracilipes

XML Treatment for Agrypon flaveolatum

XML Treatment for Agrypon flexorium

XML Treatment for Agrypon flexorioides

XML Treatment for Agrypon interstitiale

XML Treatment for Agrypon scutellatum

XML Treatment for Agrypon varitarsum

XML Treatment for Aphanistes gliscens

XML Treatment for Aphanistes klugii

XML Treatment for Aphanistes ruficornis

XML Treatment for Barylypa delictor

XML Treatment for Heteropelma amictum

XML Treatment for Heteropelma megarthrum

XML Treatment for Perisphincter gracilicornis

XML Treatment for Therion circumflexum

XML Treatment for Therion giganteum

XML Treatment for Trichomma enecator

XML Treatment for Trichomma fulvidens

XML Treatment for Trichomma occisor

## Figures and Tables

**Figure 1a.**
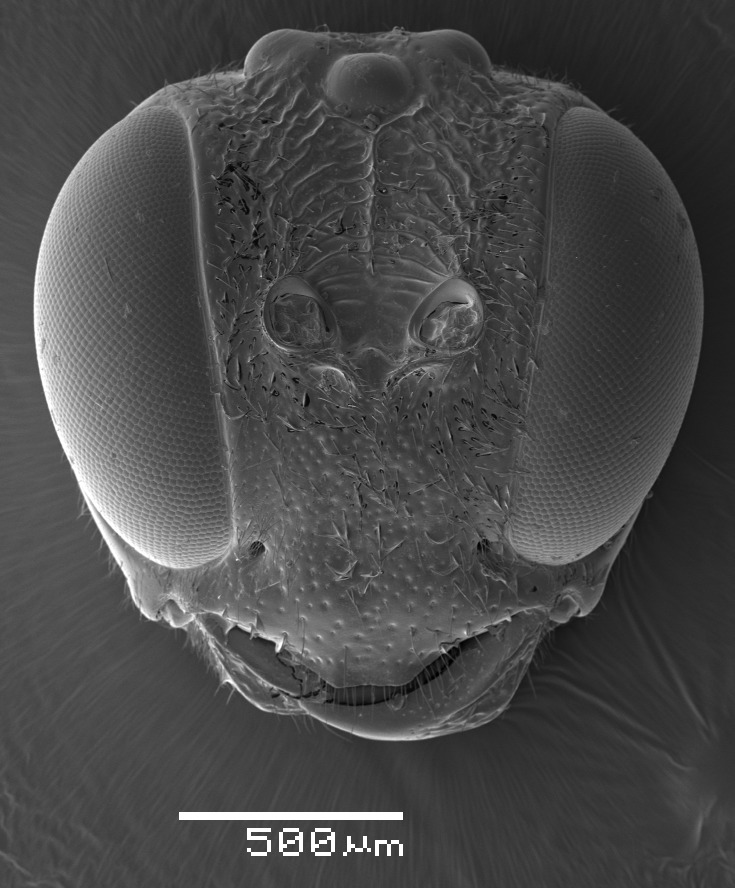
dorsal view of face

**Figure 1b.**
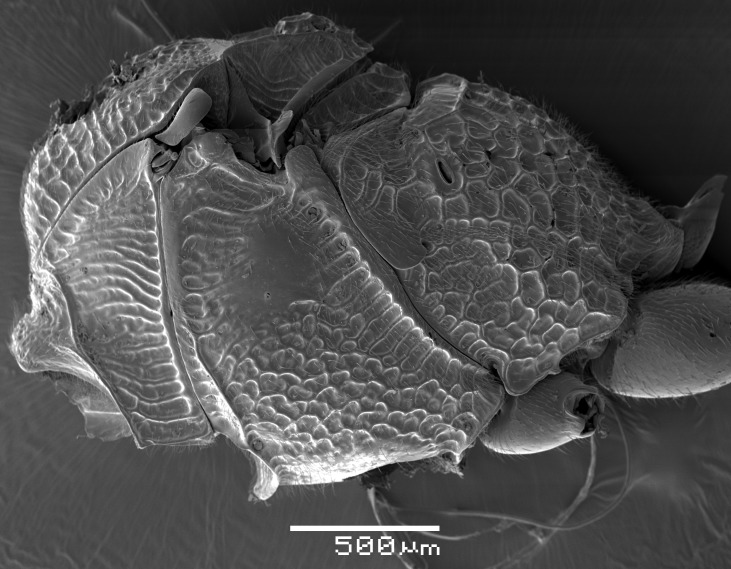
lateral view of mesosoma

**Figure 4a.**
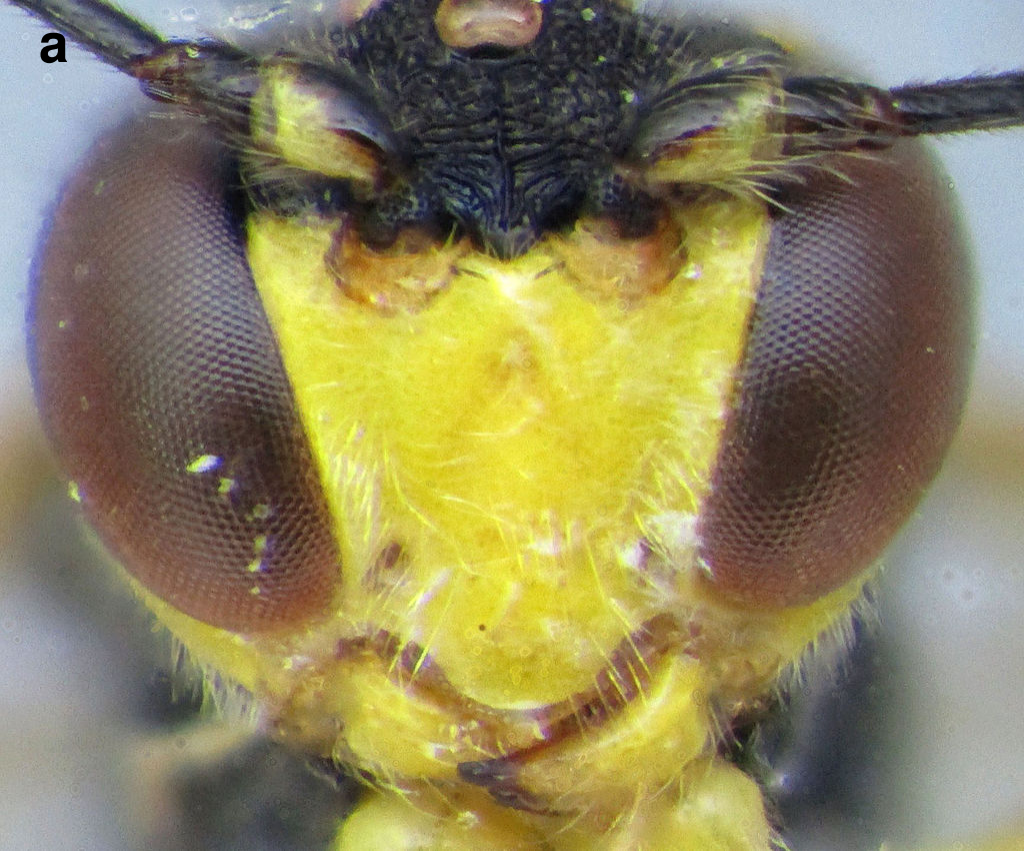
dorsal view of face

**Figure 4b.**
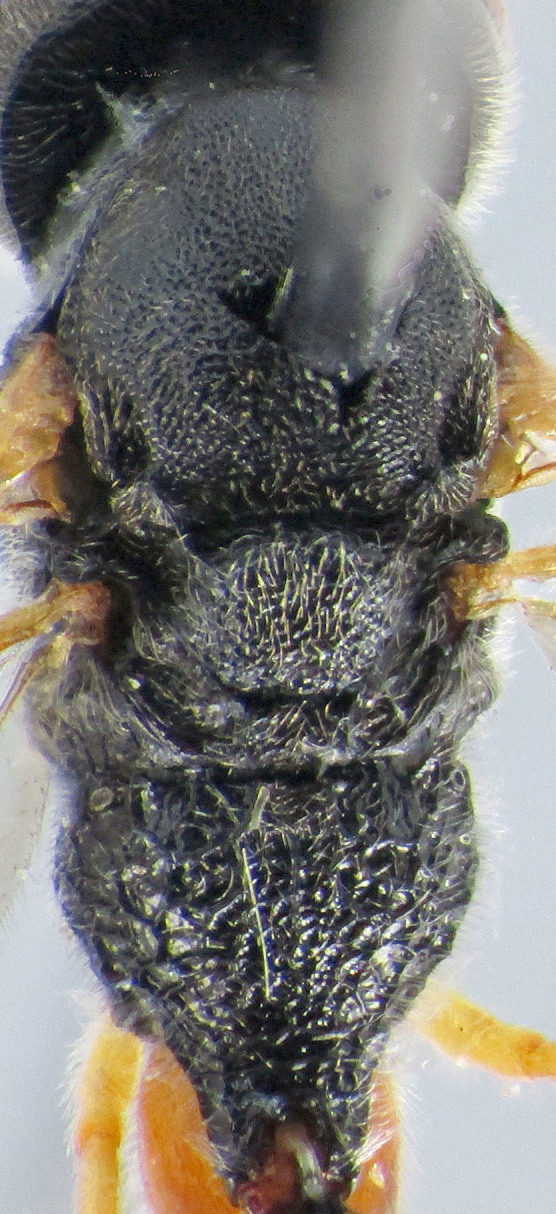
dorsal view of mesosoma

**Figure 7a.**
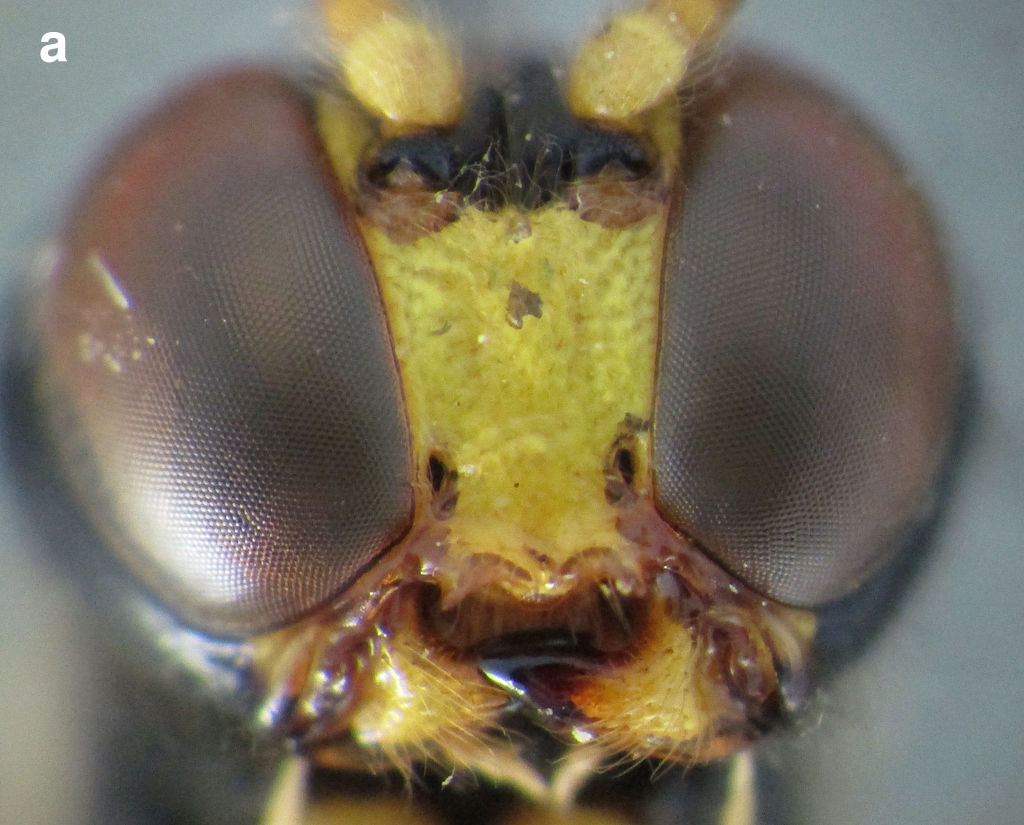
dorsal view of face

**Figure 7b.**
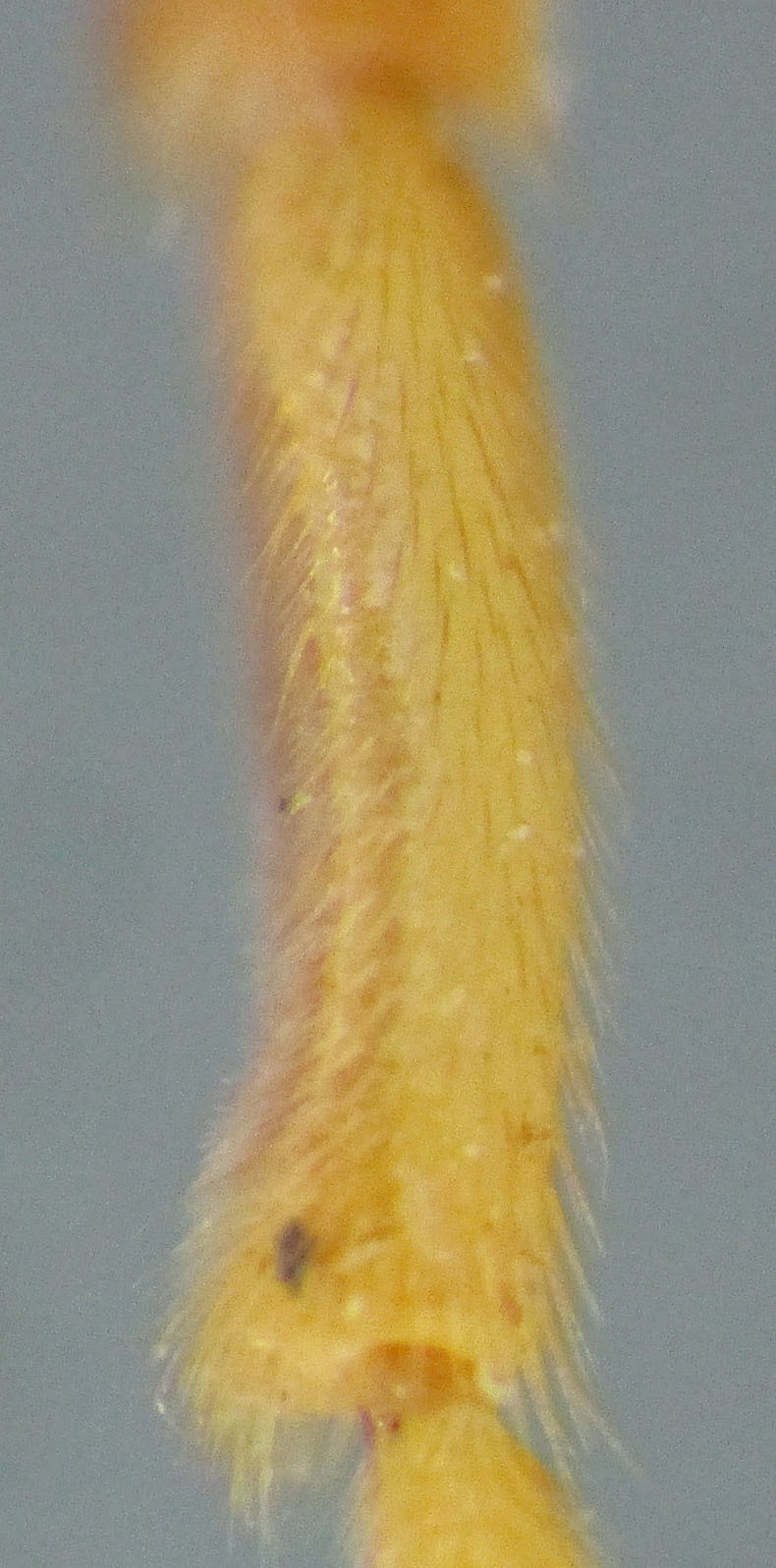
lateral view of first tarsomere of hind tarsus

**Figure 8a.**
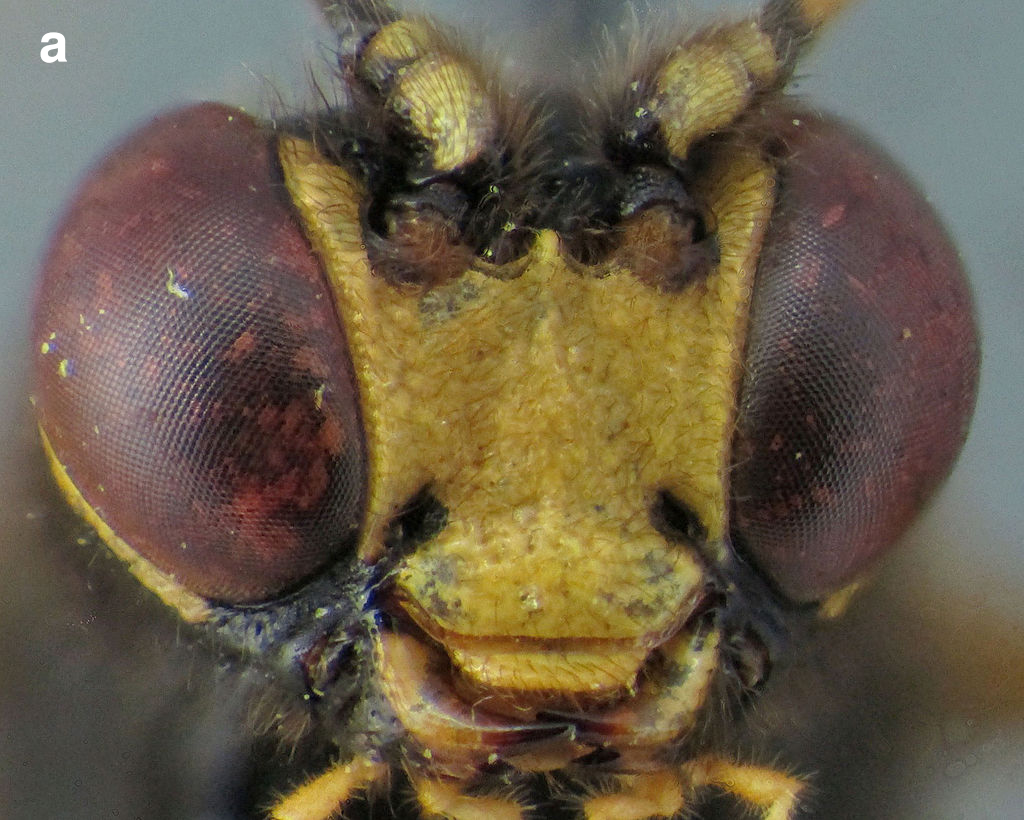
dorsal view of face

**Figure 8b.**
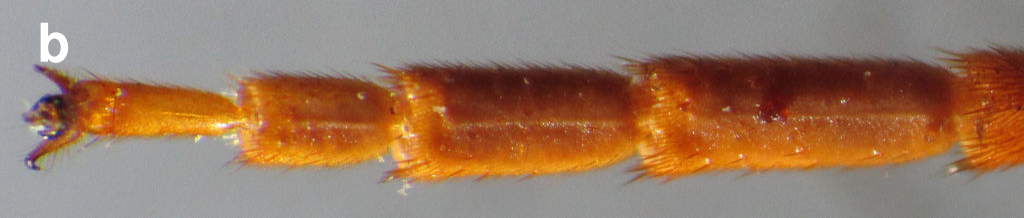
ventral view of hind tarsus

**Figure 11a.**
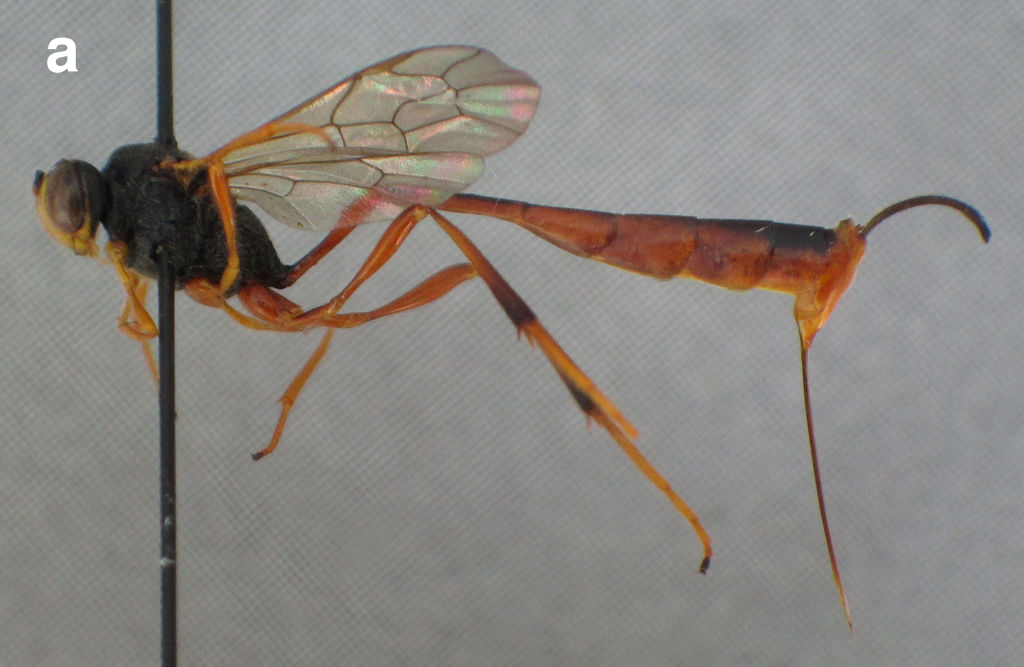
lateral view of habitus

**Figure 11b.**
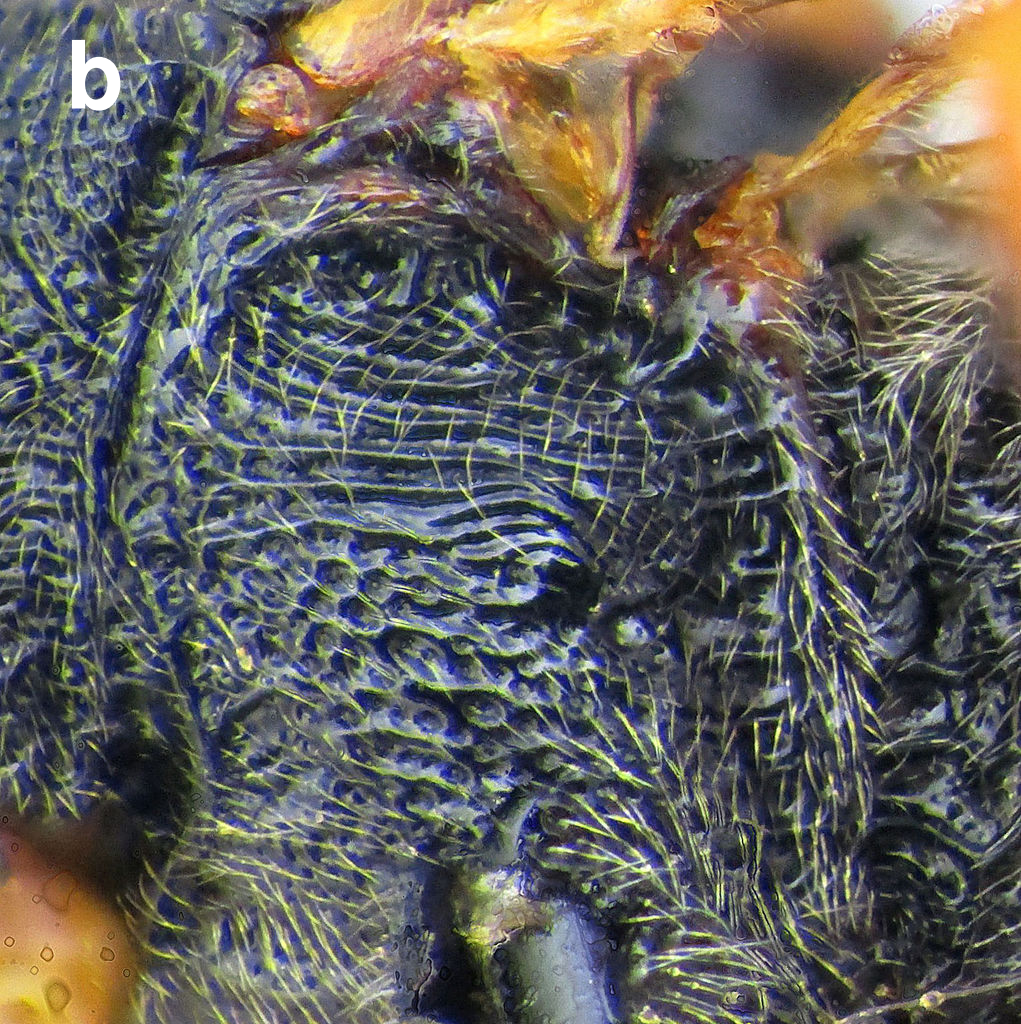
lateral view of mesopleuron

**Table 1.** High-altitude zone distribution of Anomaloninae species in the Ukrainian Carpathians.

**Species**	**foothill oak forest zone** (150–400 m a. s. l.)	**beech forest zone** (400–1300 m a. s. l.)	**coniferous borealforest zone** (900–1000 m a. s. l.)	**subalpine zone** (1400–2061 m a. s. l.)
*Anomalon cruentatum*	+			
*Agrypon anomelas*				+
*A. anxium*	+		+	+
*A. batis*	+			+
*A. clandestinum*			+	+
*A. gracilipes*	+	+		+
*A. flaveolatum*	+			+
*A. flexorium*		+	+	+
*A. flexorioides*			+	
*A. interstitiale*		+		+
*A. scutellatum*				+
*A. varitarsum*	+	+		+
*Aphanistes gliscens*	+			+
*A. klugii*	+			+
*A. ruficornis*				+
*Barylypa delictor*	+	+		
*Heteropelma amictum*	+	+	+	
*H. megarthrum*	+	+	+	
*Perisphincter gracilicornis*	+	+		
*Therion circumflexum*	+	+	+	+
*Th giganteum*			+	
*Trichomma enecator*	+	+		
*T. fulvidens*	+			
*T. occisor*	+			

**Table 2.** Appearance of Anomaloninae species adults in ten-day periods of the 1954-2014 seasons

**Species**		**April**			**May**			**June**			**July**			**Aug**			**Sep**		
		1	2	3	1	2	3	1	2	3	1	2	3	1	2	3	1	2	3
*Anomalon cruentatum*																			
*Agrypon anomelas*																			
*A. anxium*																			
*A. batis*																			
*A. clandestinum*																			
*A. gracilipes*																			
*A. flaveolatum*																			
*A. flexorium*																			
*A. flexorioides*																			
*A. interstitiale*																			
*A. scutellatum*																			
*A. varitarsum*																			
*Aphanistes gliscens*																			
*A. klugii*																			
*A. ruficornis*																			
*Barylypa delictor*																			
*Heteropelma amictum*																			
*H. megarthrum*																			
*Perisphincter gracilicornis*																			
*Therion circumflexum*																			
*Th giganteum*																			
*Trichomma enecator*																			
*T. fulvidens*																			
*T. occisor*																			
